# A novel benzimidazole derivative, MBIC inhibits tumor growth and promotes apoptosis via activation of ROS-dependent JNK signaling pathway in hepatocellular carcinoma

**DOI:** 10.18632/oncotarget.14606

**Published:** 2017-01-12

**Authors:** Xiaoyun Dai, Lingzhi Wang, Amudha Deivasigamni, Chung Yeng Looi, Chandrabose Karthikeyan, Piyush Trivedi, Arunachalam Chinnathambi, Sulaiman Ali Alharbi, Frank Arfuso, Arunasalam Dharmarajan, Boon Cher Goh, Kam Man Hui, Alan Prem Kumar, Mohd Rais Mustafa, Gautam Sethi

**Affiliations:** ^1^ Department of Pharmacology, Yong Loo Lin School of Medicine, National University of Singapore, Singapore; ^2^ Cancer Science Institute of Singapore, Centre for Translational Medicine, Singapore; ^3^ Division of Cellular and Molecular Research, Humphrey Oei Institute of Cancer Research, National Cancer Centre, Singapore; ^4^ Department of Pharmacology, Faculty of Medicine, University of Malaya, Kuala Lumpur, Malaysia; ^5^ School of Pharmaceutical Sciences, Rajiv Gandhi Proudyogiki Vishwavidyalaya, Bhopal, India; ^6^ Department of Botany and Microbiology, College of Science, King Saud University, Riyadh, Kingdom of Saudi Arabia; ^7^ Stem Cell and Cancer Biology Laboratory, School of Biomedical Sciences, Curtin Health Innovation Research Institute, Curtin University, Perth WA, Australia; ^8^ Department of Haematology-Oncology, National University Health System, Singapore; ^9^ Institute of Molecular and Cell Biology, A*STAR, Biopolis Drive Proteos, Singapore; ^10^ Cancer and Stem Cell Biology Program, Duke–National University of Singapore Graduate Medical School, Singapore; ^11^ Department of Biochemistry, Yong Loo Lin School of Medicine, National University of Singapore, Singapore; ^12^ Curtin Medical School, Faculty of Health Sciences, Curtin University, Perth WA, Australia; ^13^ Department of Biological Sciences, University of North Texas, Denton, Texas, USA; ^14^ School of Biomedical Sciences, Curtin Health Innovation Research Institute, Curtin University, Perth WA, Australia

**Keywords:** MBIC, HCC, JNK, ROS, apoptosis

## Abstract

A prior screening programme carried out using MTT assay by our group identified a series of novel benzimidazole derivatives, among which Methyl 2-(5-fluoro-2-hydroxyphenyl)-1H- benzo[d]imidazole-5-carboxylate (MBIC) showed highest anticancer efficacy compared to that of chemotherapeutic agent, cisplatin. In the present study, we found that MBIC inhibited cell viability in different hepatocellular carcinoma (HCC) cell lines without exerting significant cytotoxic effects on normal liver cells. Annexin V-FITC/PI flow cytometry analysis and Western blotting results indicated that MBIC can induce apoptosis in HCC cells, which was found to be mediated through mitochondria associated proteins ultimately leading to the activation of caspase-3. The exposure to MBIC also resulted in remarkable impairment of HCC cell migration and invasion. In addition, treatment with MBIC led to a rapid generation of reactive oxygen species (ROS) and substantial activation of c-Jun-N-terminal kinase (JNK). The depletion of ROS by N-Acetyl cysteine (NAC) partially blocked MBIC-induced apoptosis and JNK activation in HCC cells. Finally, MBIC significantly inhibited tumor growth at a dose of 25 mg/kg in an orthotopic HCC mouse model. Taken together, these results demonstrate that MBIC may inhibit cell proliferation via ROS-mediated activation of the JNK signaling cascade in HCC cells.

## INTRODUCTION

Hepatocellular carcinoma (HCC) is one of the most common and lethal cancers in the world, especially in men, and it is the second leading cause of cancer death in developing countries [1]. The majority of patients with HCC are diagnosed at an intermediate or advanced stage [2]. For patients with advanced HCC, systemic chemotherapy with small kinase inhibitors or cytotoxic agents provides marginal benefit; for example, sorafenib, an oral multi-tyrosine kinase inhibitor, is a standard of care for these patients, but the median overall survival for the patients treated with it is only around three months longer than for patients treated with placebo [3, 4]. Furthermore, HCC patients with multiple lung metastases have a poor prognosis with no efficacious treatment being identified till now [5]. Therefore, it is imperative to develop novel pharmacological agents for the treatment of HCC.

Reactive oxygen species (ROS) play an essential role in cellular proliferation, differentiation, and apoptosis. In cancer therapy, majority of chemotherapeutics and radiotherapeutics destroy cancer cells by generating substantial quantity of ROS [6, 7]. The imbalance between the levels of oxidizing and reducing equivalents generates a high concentration of ROS that may lead to cell death [8]. ROS may act as intracellular messengers or alter the protein structure and function by oxidizing critical amino acid residues [9]. Several prior studies have described that ROS can mediate the sustained activation of the mitogen-activated protein kinases (MAPK) pathways, which play a critical role in various physiological processes [10]. In human cells, the MAPK pathways consist of three different protein kinases including the extracellular signal-regulated kinases (ERKs), the p38 MAPKs, and the c-Jun N-terminal kinases (JNKs) [11]. Generally, ERK cascades are often activated by growth factors and survival factors that are associated with cell survival. In contrast, the JNK and p38 MAPK pathways are activated by stress stimuli that are frequently associated with pro-apoptotic effects [3, 12]. Interestingly, diverse apoptotic stimuli such as ROS and tumor necrosis factor TNF-α can induce apoptosis signal-regulated kinase (ASK)1 activation, eventually resulting to the activation of JNK signalling cascade and apoptosis [13, 14]. Numerous studies have described that JNK activation may initiate apoptotic signalling via two different strategies. The first strategy is to directly activate mitochondrial pro- and anti-apoptotic proteins such as Bcl-2 proteins [15], whereas as another indirect strategy can be mediated via the rapid translocation of the JNK to the nucleus where it phosphorylates and regulates the activity of transcription factors such as c-Jun, which further upregulates pro-apoptotic genes [16, 17].

**Figure 1:**
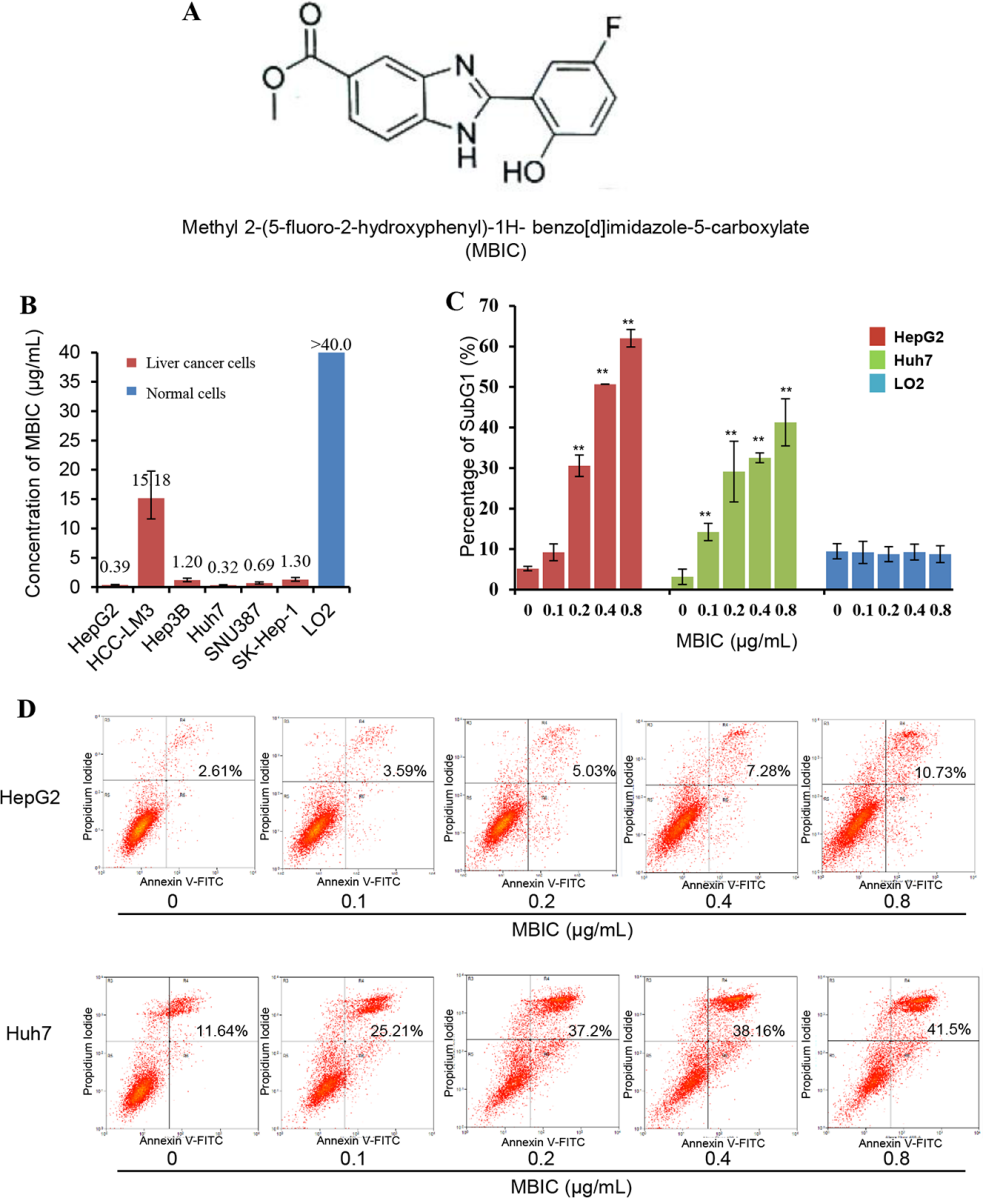
The effect of MBIC on suppression of cell viability and the induction of apoptosis in HCC cells (**A**) The chemical structure of MBIC. (**B**) MTT assay was performed to determine IC_50 values_ upon exposure to different concentrations of MBIC for 72 h. (**C**) SubG1 percentage was determined by Propidium Iodide staining. Cells were treated with MBIC at 0.1, 0.2. 0.4 or 0.8 μg/ml for 72 h. After staining with Propidium Iodide, the cells were analyzed using a flow cytometer. Error bars are means ± SD. Ordinary one-way ANOVA with Dunnett's Multiple Comparison Test (**p < 0.01). (**D**) The Annexin V and PI apoptosis assay demonstrating the increase in Annexin V-PI positive cell populations with increasing dose of MBIC.

Benzimidazoles are heterocyclic aromatic compounds that contained a fusion of a phenyl and imidazole ring [18]. Benzimidazoles and their derivatives possess many important pharmacological properties; for example omeprazole (proton pump inhibitor), pimobendan (ionodilator), and mebendazole (anthelmintic) [19, 20]. Benzimidazole-derived scaffolds are gaining attention in medicinal chemistry because of the presence of a heterocyclic imidazole ring that provides excellent possibilities for generating potential anti-cancer agents [21]. In search of novel anti-cancer drugs, we synthesized a series of benzimidazole derivatives and screened them against a panel of human cancer cell-lines. One promising hit, Methyl 2-(5-fluoro-2-hydroxyphenyl)-1H- benzo[d]imidazole-5-carboxylate (MBIC) (Figure 1A), showed potent anti-proliferative activity compared to the chemotherapeutic drug cisplatin [22]. In the present study, the possible molecular mechanism(s) underlying the anticancer effects of MBIC were investigated in diverse HCC cell lines. It was observed that MBIC-induced apoptosis might be mediated by the ROS-induced activation of JNK signaling in HCC cells that led to the inhibition of tumor growth in an orthotopic mouse model.

## RESULTS

### MBIC inhibits viability and induces substantial apoptosis in diverse HCC cell lines

The effect of MBIC on cellular viability was analyzed by MTT assay in various HCC cells. As shown in Figure 1B, MBIC was highly cytotoxic towards HepG2, Hep3B, Huh7, SNU387, and SK-Hep-1 cells, with IC_50_ values of 0.39, 1.20, 0.32, 0.69, and 1.30 μg/mL respectively at 72 h. Comparatively, HCCLM3, a highly metastatic cell line, was more resistant to MBIC treatment as compared to other HCC cells [23], and its IC_50_ value was around 15.18 μg/mL. A parallel study was performed to determine the effect of MBIC against normal liver cells. No significant inhibition was found in normal liver cell line, LO2 cells after treatment with MBIC for 72 h, and the IC_50_ value was found to be higher than 40 μg/mL.

The apoptotic potential of MBIC was measured by flow cytometry after staining with PI. After treatment for 72 h, induction of apoptosis in both HepG2 and Huh7 was dramatically increased with increasing concentrations of MBIC from 0.1 to 0.8 μg/mL (Figure 1C), which was consistent with the MTT results. The percentage of SubG1 cells in the normal LO2 hepatic cell line was always less than 10% at the different concentrations of MBIC for 72 h. Next, PI-Annexin V double staining was used to further identify the effects of MBIC on cellular apoptosis. Interestingly, PI-Annexin V double positive population, which indicates the later stage of apoptosis, also increased in a dose-dependent manner after treatment with MBIC for 72 h in both HepG2 and Huh7 cells (Figure 1D). These results suggest that MBIC can significantly inhibit the growth of HCC cells and induce substantial apoptosis in a dose-dependent manner, while being significantly less cytotoxic towards normal liver cells.

### MBIC-induces apoptosis through caspase-mediated pathway

**Figure 2:**
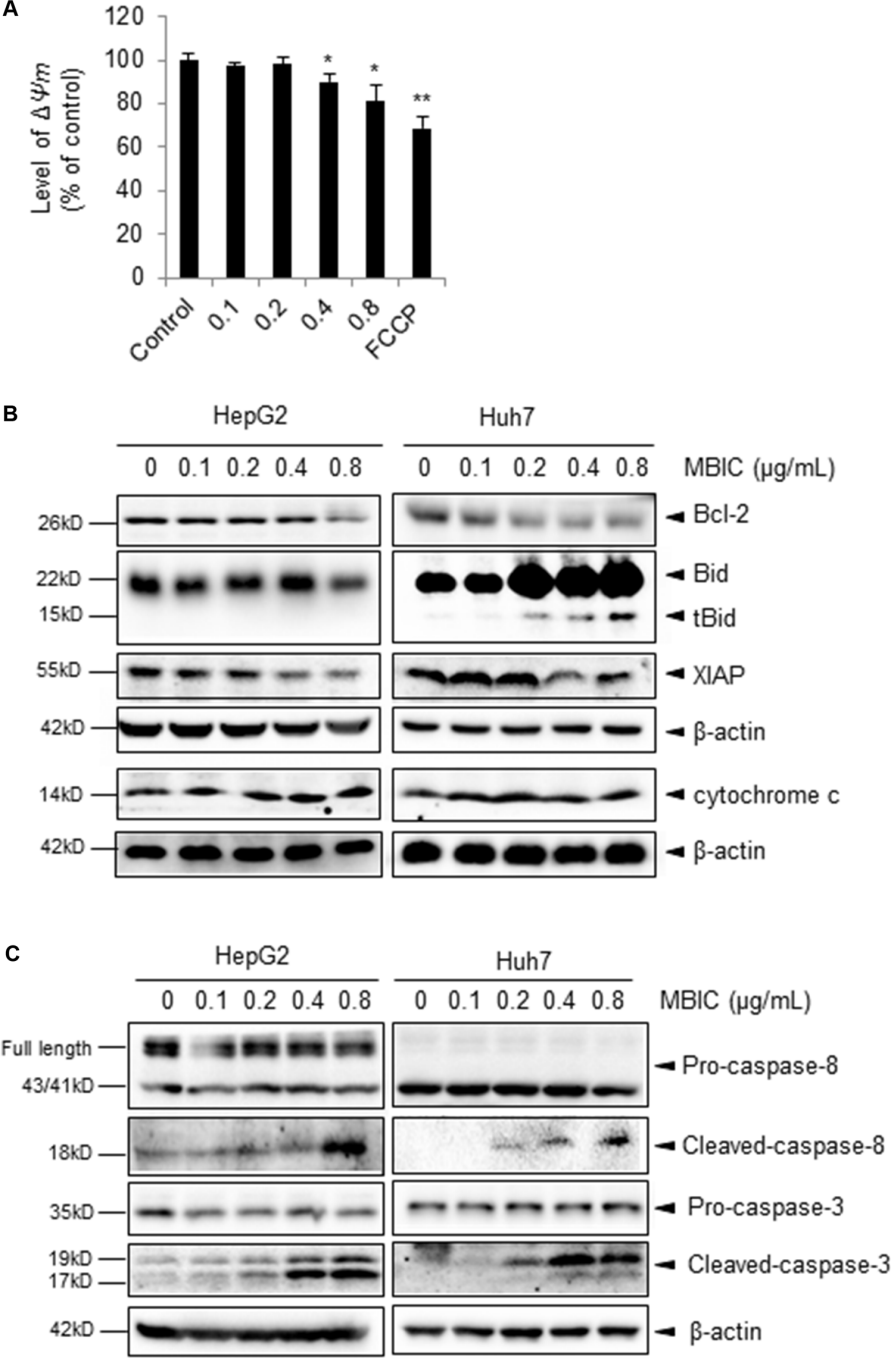
The potential effect of MBIC on the cellular apoptotic pathways (**A**) The mitochondrial potential level was determined by staining with TMRE. Huh 7 cells were treated with various concentrations of MBIC for 24 h and then stained by 1 μΜ TMRE. 10 μM FCCP was added as a positive control. Error bars are means ± SD *n* = 2. Ordinary one-way ANOVA with Dunnett's Multiple Comparison Test (*p < 0.05; **p < 0.01). (**B**) The expression of various proteins was determined by Western blot analysis after treatment with indicated concentrations of MBIC for 24 h. (**C**) Caspase family proteins were analysed by Western blotting.

It has been reported that mitochondria and caspases are the central executioners of apoptosis [24]. To investigate the contribution of mitochondria in MBIC-induced apoptosis of HCC cells, the mitochondrial membrane potential (Δψ*m*) was measured by staining cell with TMRE. As shown in Figure 2A, the Δψ*m* was significantly decreased after exposure to 0.4 and 0.8 μg/mL of MBIC for 12 h. Next, we investigated the protein levels of various pro-apoptotic proteins by Western blot analysis. The results shown in Figure 2B indicate that protein levels of Bcl-2 and XIAP were decreased and cytochrome c level was increased in HepG2 and Huh7 cells after treatment with the MBIC for 24 h. In addition, the expression of active form, tBid, was triggered in a dose-dependent manner in MBIC-treated Huh7 cells although full length Bid level was found to be increased for unclear reasons that require further analysis. To determine the contribution of caspases to the apoptotic process, the level of caspase family proteins was assessed by Western blotting analysis. As shown in Figure 2C, MBIC induced the cleavage of caspase-8 and caspase-3 in a dose-dependent manner in HCC cells. Taken together, the data suggest that MBIC-induced apoptosis might be mediated by caspase-dependent process.

### MBIC inhibits cellular migration and invasion in HCC cells

**Figure 3:**
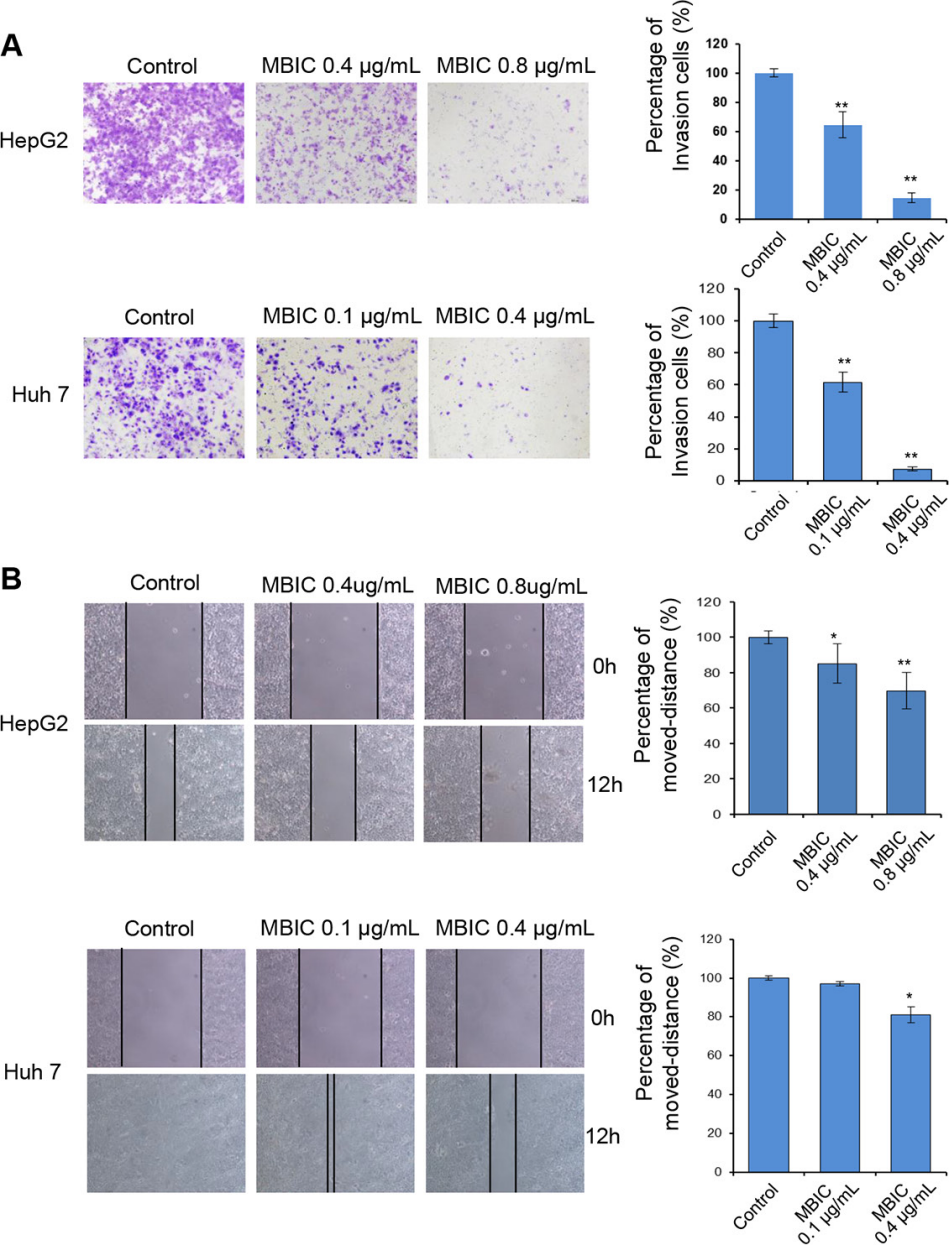
MBIC abrogates cellular migration and invasion (**A**) HepG2 and Huh7 cells were either untreated or treated with MBIC for 12 h and subjected to the invasion (with Matrigel) assays. Invasive cells were imaged by bright-field microscope under × 100 magnification. Representative images are shown for indicated concentrations of MBIC, and results were quantified using Image J software. (**B**) Scratch wound-healing assay performed in HepG2 and Huh7 cells with or without MBIC treatment. Results are also quantified using Image J software. (Magnification × 100). Error bars are means ± SD. Ordinary one-way ANOVA with Dunnett's Multiple Comparison Test (*p < 0.05; **p < 0.01).

We next examined whether MBIC could also affect the migratory and invasive behavior of HCC cells. The chamber invasion assay was performed to investigate cellular invasive ability. As shown in Figure 3A, the results indicated that MBIC significantly reduced the infiltration rates of HepG2 and Huh7 cells compared to the untreated control cells at two different concentrations. Meanwhile, the effect of MBIC on Huh7 cells was found to be more effective than HepG2 at the 0.4 μg/ml. In addition, the scratch wound healing assay also demonstrated similar results for HepG2 and Huh7 cells, indicating that MBIC could significantly reduce the migration of both HCC cell lines (Figure 3B). These results cumulatively suggest that MBIC could exert an inhibitory effect on both cellular migration and invasion of HCC cells.

### MBIC activates ROS-dependent JNK signaling pathway

**Figure 4:**
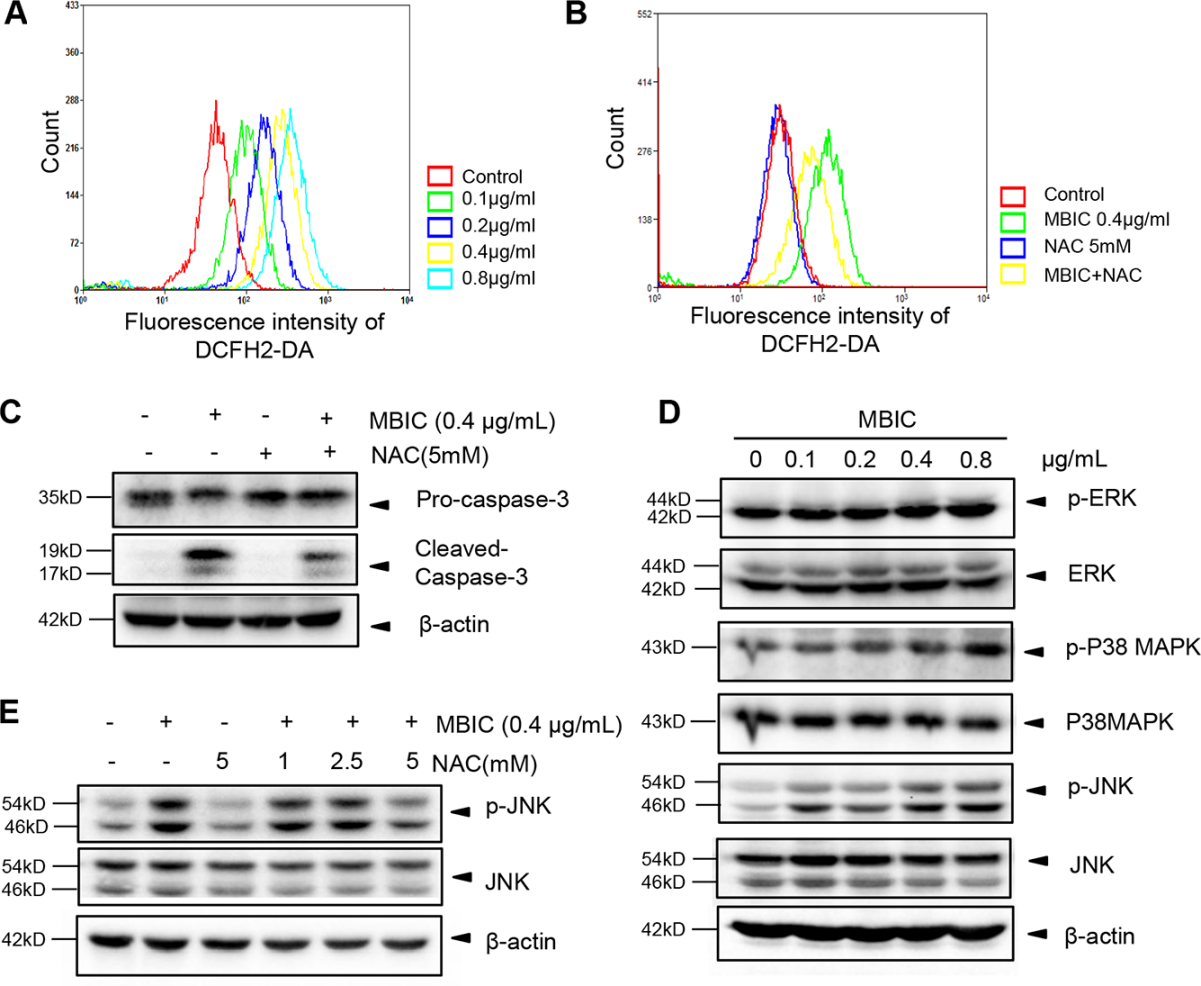
MBIC activates JNK signaling pathway via ROS generation (**A**) The quantity of ROS were analysed by H2DCF-DA staining after treatment with MBIC for 2 h. (**B**) Huh7 cells were exposed to 0.4 μg/mL MBIC with or without NAC (5 mM) for 2 h, and then ROS production was analyzed by staining with H2DCF-DA. (**C**) Huh7 cells were exposed to 0.4 μg/mL MBIC with or without NAC (5 mM) for 24 h. The expression of cleaved-caspase-3 was determined by Western blotting analysis. (**D**) Huh7 cells were treated with various concentrations of MBIC for 8 h. The ERK, P38, and JNK phosphorylation was determined by Western blotting analysis. (**E**) Huh7 cells were exposed to 0.4 μg/mL MBIC with or without the indicated concentrations of NAC for 2 h. The activation of JNK was determined by Western blotting analysis.

Numerous studies have demonstrated that ROS production can significantly induce apoptosis [25]. To investigate whether ROS generation is one of the upstream molecular events involved in MBIC-induced apoptosis, we used flow cytometry analysis to examine ROS generation with the fluorescent probe 2,7-dichlorodihydrofluorescein diacetate (DCFH2-DA). As shown in Figure 4A, the rapid generation of ROS was detected at 2 h following MBIC treatment. Compared with the control, about 8-fold change was found after exposure to 0.8 μg/mL MBIC for 2 h. Previous reports also have indicated that N-acetyl cysteine (NAC), a ROS scavenger, can prevent apoptosis and promote cell survival [26, 27]. Interestingly, it was noted that the effect of MBIC on ROS production was partially reversed by pre-treatment with 5 mM NAC for 2 h (Figure 4B). Furthermore, as shown in Figure 4C, NAC also partially abolished the MBIC-induced cleaved-caspase-3 expression, which plays a central role in the execution-phase of apoptosis.

It has been reported that ROS could induce the phosphorylation and sustained activation of MAPK signaling proteins such as ERK, p38, and JNK [28]. In order to investigate whether MBIC-induced ROS production leads to the activation of MAPK signaling proteins, we employed Western blot analysis to examine the effect of MBIC on phosphorylation status of these proteins. As shown in Figure 4D, no obvious change of phospho-ERK and a slight increase in phospho-p38 MAPK were observed upon MBIC treatment in HCC cells. However, the expression of p-JNK was substantially up-regulated in a dose-dependent manner after MBIC treatment. To further investigate the mechanisms underlying ROS-dependent JNK activation, we determined the effect of NAC on the JNK phosphorylation that can be induced upon MBIC treatment. As shown in Figure 4E, Western blotting results showed that the MBIC-induced JNK activation could also be abrogated by NAC in a dose-dependent manner. Altogether, all these results indicated that MBIC-induced apoptosis might be mediated through a ROS-dependent JNK signaling pathway.

### MBIC did not produce any significant toxic effects in mice

**Figure 5:**
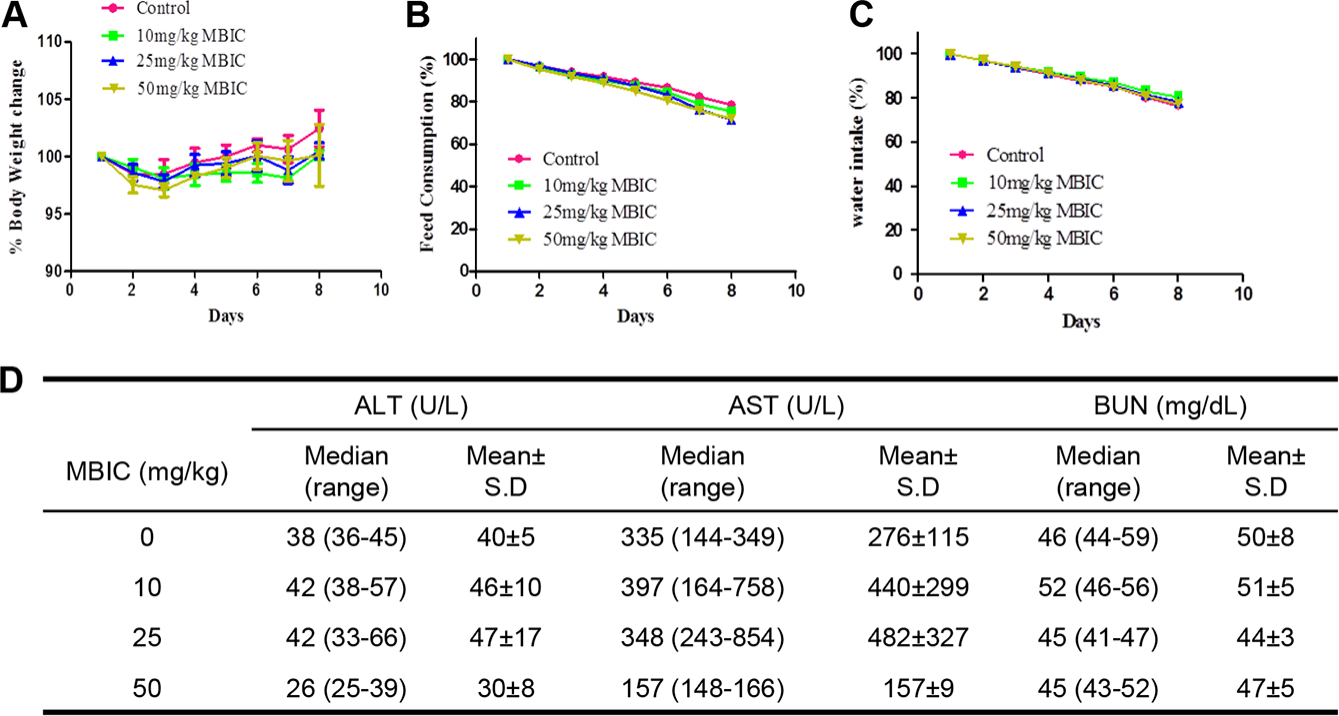
Acute toxicity studies with MBIC (**A**) The effect of intraperitoneal administration of MBIC on body weight change. The nude mice were treated with one single dose of MBIC (10, 25, or 50 mg/kg). Error bars are means ± SD. Ordinary one-way ANOVA with Dunnett's Multiple Comparison Test. (**B**–**C**) The effect of MBIC on mice behavior study including feed consumption (B) and water intake (C). Error bars are means ± SD. Ordinary one-way ANOVA with Dunnett's Multiple Comparison Test. (**D**) Effect of MBIC on biochemical parameters.

We next carried out acute toxicity studies with MBIC to determine its sub-lethal dose for *in vivo* studies. The mice were monitored for 8 days after the intraperitoneal administration of the 10, 25, or 50 mg/kg dose of MBIC and vehicle (0.1% DMSO). At the end of the experiment, no mortality was observed, which indicated that the LD_50_ must be higher than 50 mg/kg. As shown in the Figure 5A–5C, there was no significant difference of behavioral and physical symptoms such as body weight, feed consumption, and water intake between MBIC treated groups and the control group.

The various biochemical parameters of the serum including alanine aminotransferase (ALT), aspartate aminotransferase (AST), and blood urea nitrogen (BUN) were detected by the autoanalyzer. It was observed that compared to the control group, no substantial differences were found in the serum levels of ALT, AST, and BUN in the MBIC-treated groups. All these results indicate that the intraperitoneal administration of MBIC did not produce any obvious toxic effects in NCr nude mice (Figure 5D).

### MBIC inhibited tumor growth in an orthotopic HCC mouse model

**Figure 6:**
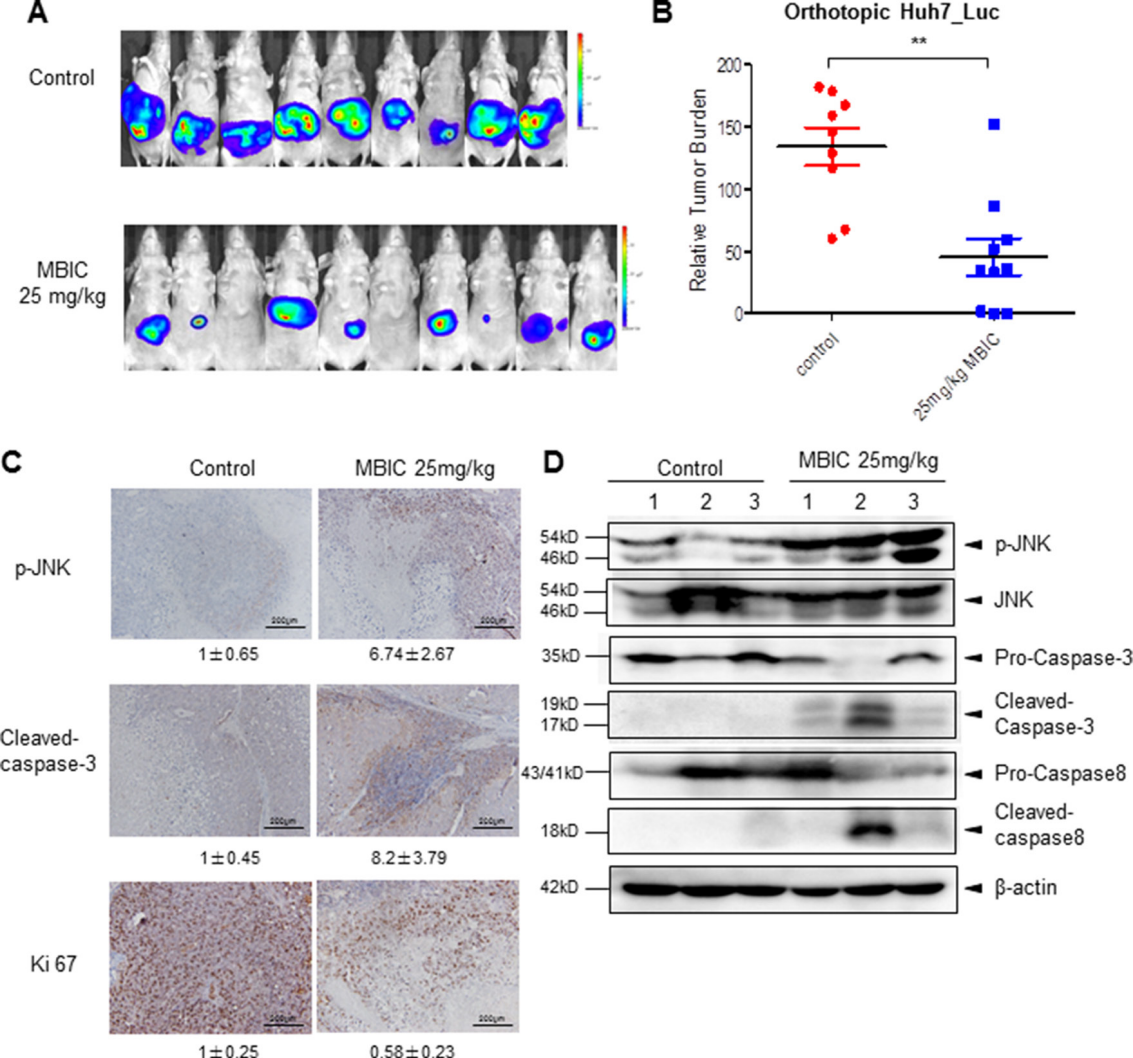
MBIC significantly inhibits tumor growth in an orthotopic mouse model (**A**) Bioluminescence images of orthotopically implanted tumours. Ncr nude mice were orthotopically implanted with Huh 7_Luc cell-induced tumors and then treated with 0.1% DMSO (*n* = 9) or 25 mg/kg MBIC (*n* = 10) for 29 days. (**B**) The scatter plot represents the differences in tumor burden at the last point by measuring photon counts and is expressed as tumor burden relative to photon counts before first therapeutic injection. Unpaired *t* test with Welch's correction (**p < 0.01). (**C**) Immunohistochemistry showing p-JNK, cleaved-caspase-3, and Ki 67 levels in tumors from vehicle control or 25 mg/kg MBIC-treated mice. Fold change of positive staining for the biomarkers was shown. Magnification 200 ×. (**D**) The western blot analysis of p-JNK and caspase family proteins in tumor tissues. At least three samples were analyzed from each group.

We also analyzed the anti-tumor potential of MBIC *in vivo* via intraperitoneal administration using the Huh7_Luc orthotopic model. After treatment with 25 mg/kg of MBIC (three doses per week for 4 consecutive weeks), bioluminescence images revealed that there was a significant reduction of tumor growth in the MBIC group compared with the vehicle control group at the end of the assay (Figure 6A). The differences in tumor burden at the last point was quantitated by measuring photon counts and expressed as the tumor burden relative to photon counts before the first therapeutic injection. An unpaired *t* test with Welch's correction indicated that the MBIC treatment group had significant inhibition of tumor burden compared with the vehicle-treated controls (Figure 6B).

We further evaluated the effect of MBIC on the expression level of p-JNK in tumor tissues by immunohistochemistry and found that MBIC substantially increased JNK phosphorylation in drug treated group as compared with the control group. Consistently, the expression of cleaved-caspase-3 staining in HCC tumor tissues was also increased after MBIC treatment, especially in cells present in the close vicinity to the blood vessels. Moreover, the expression of proliferative biomarker, Ki67 was down-regulated in treated group as compared to the control group (Figure 6C). The protein levels of p-JNK, cleaved-caspase-3, and cleaved-caspase-8 in tumor tissues were further investigated by Western blotting analysis, and the results also showed a marked increase in expression of these proteins in the MBIC treatment group (Figure 6D). Overall, these results indicate that MBIC exerts its anticancer effects by modulating the expression of diverse biomarkers associated with tumor growth and survival.

## DISCUSSION

We had previously prepared various substituted 2-(phenyl)-3*H*-Benzo[*d*]Imidazole-5-carboxylic acids and their methyl esters and then screened them for their anti-proliferative effects against breast cancer cell lines [22]. Although benzo [*d*]imidazole-5-carboxylic acids showed only modest effect, the compound MBIC with 2-hydroxyl and 5-fluoro substitution in the aryl ring was found to exert the most potent anti-proliferative effects against breast cancer cells. In this study, we performed MTT assay to demonstrate that MBIC could significantly inhibit cell viability in a panel of HCC cells, especially in HepG2 and Huh7 cells, with IC_50_ values of 0.39 and 0.32 μg/mL respectively. Interestingly, no obvious cytotoxic effect of the drug was observed on normal liver cells in which the IC_50_ was found to be higher than 40 μg/mL. These results suggested that MBIC specifically inhibited growth potential of liver cancer cells, but had no detrimental effect on normal liver cells.

The induction of cellular apoptosis constitutes the main strategy for cancer treatment [29]. Here, we used an Annexin V-PI staining assay to reveal that MBIC induced apoptosis of HCC cells. To examine the molecular mechanism(s) underlying this MBIC-induced apoptosis, we analyzed the changes of mitochondrial membrane potential, which is an important step in initiating intrinsic apoptotic pathway [30]. Our results indicated that MBIC significantly decreased mitochondrial membrane potential in HCC cells. The loss of the mitochondrial membrane potential mediated by proteins from the Bcl-2 family, could lead to the release of apoptogenic factors such as cytochrome c into the cytoplasm. This in turn trigger activation of caspase-9 and caspase-3, which play a pivotal role in the execution of apoptosis [31]. The down-regulation of anti-apoptotic Bcl-2 and up-regulation of pro-apoptotic Bid proteins was found after treatment with MBIC for 24 h. Meanwhile, MBIC also increased cytochrome c and cleaved-caspase-3 expression. These results suggest that the intrinsic apoptotic pathway may be predominantly involved in the MBIC-induced apoptosis. The extrinsic apoptotic pathway can be activated by cell surface death receptors, such as TNF-related apoptosis-inducing ligand (TRAIL), leading to the activation of caspase-8 [32]. An apparent induction of cleaved caspase-8 was also detected, indicating that MBIC-induced apoptosis may also involve extrinsic pathway to some extent in HCC cells. However, the exact roles of both intrinsic and extrinsic pathways in MBIC-induced apopotosis requires additional detailed investigations.

An excessive ROS production leads to disruption of intracellular redox homeostasis, which can induce cellular apoptosis through the both intrinsic and extrinsic pathways [33]. It is well documented that excessive production of ROS may activate MAPK signalling cascades such as JNK pathway, which plays an important role in many cellular events [16]. A recent report has identified that another new benzimidazole acridine derivative, N-{(1H-benzo[d]imidazol-2-yl)methyl}-2-butylacridin-9-amine, can induce apoptosis via the ROS-JNK signaling pathway in human colorectal cancer cell lines [34]. Consistent with this study, MBIC was also found to induce a substantial increase of ROS production and JNK phosphorylation in HCC cells. This ROS production was found to be partially abrogated upon NAC treatment. Because oxidative stress can be controlled by different antioxidant mechanisms such as antioxidant modulators and enzymatic scavengers [35], our results suggest that diverse mechanism(s) may be involved in MBIC-induced ROS production. Taken together, our findings suggest that the ROS-JNK pathway may be involved in MBIC-induced apoptosis. The exact apoptosis relationship between MBIC-mediated ROS production, apoptosis and activation of JNK signal transduction requires further investigation.

In conclusion, our study is the first to demonstrate that MBIC can induce substantial apoptosis mediated by the ROS/JNK signalling pathway and significantly abrogate tumor growth in an orthotopic mouse model without exhibiting any major adverse effects. The findings of this study expand the potential understanding of molecular mechanisms(s) mediating anticancer effects of MBIC to a great extent and support further evaluation of its clinical efficacy.

## MATERIALS AND METHODS

### Synthesis of MBIC

The detailed protocol for the synthesis of MBIC was well-described in a previous published paper [22]. The chemical structure of the synthesized compound was confirmed using different spectroscopic techniques, including ^1^H NMR, IR, and LC–MS analyses and elemental analysis.

### Cell lines

HepG2, Hep3B, SNU387, SK-Hep-1, and LO2 cell lines were obtained from American Type Culture Collection. Huh7 and HCCLM3 cell lines were a kind gift from Professor Zhao-You Tang at the Liver Cancer Institute (Zhongshan Hospital, Fudan University, Shanghai). All the HCC cells were cultured in Dulbecco's Modified Eagle Medium (DMEM) with 10% FBS.

### MTT assay

HCC cells (4~7 × 10^3^) were seeded in 96-well plates, and then treated with indicated concentrations of MBIC for 72 h. After treatment, the cells were incubated with 20 μL 5 mg/mL MTT for 2 to 4 hours until a purple precipitate was visible. Thereafter, 0.1 mL lysis buffer (20% SDS, 50% dimethylformamide) was added after removal of the medium, and then incubated at 37°C for 1 h. The absorbance was measured at 570 nm using a Tecan plate reader.

### Wound healing assay

An Ibidi Culture–Insert (Ibidi GmbH, Germany) was used for the scratch wound healing assays. HCC cells (7 × 10^5^) were seeded in the Culture-Insert to form a confluent layer within 24 hours, and then treated with different concentrations of MBIC for 8 h. A cell-free gap of 500 μm was created after gently removing the Culture-Insert. The wounds were observed using microscopy (Olympus DP 70, Japan) after migration for 24 h and the gap closure was measured.

### Invasion assay

HCC cells were seeded in a Bio-Coat Matrigel invasion chamber (BD, USA) with FBS-free medium overnight. After incubation with MBIC for 8 h, the medium was changed to DMEM medium with 10% FBS. After invasion for 24 h, the inserts were stained by the 1% crystal violet and observed using bright field microscopy (Olympus DP 70, Japan).

### PI staining

Treated cells were harvested by trypsin without EDTA, washed by PBS, and fixed with 70% ethanol for 30 mins. The cells were stained with propidium iodide solution, and intensity of fluorescence was measured by flow cytometry (BD, Biosciences, San Jose, CA) using the FL2-PI channels.

### Annexin V-PI staining

Apoptosis was assessed by Annexin V-PI staining (Santa cruz Co, CA). After treatment, the cells were harvested by trypsin without EDTA, and washed using ice-cold PBS. The cells were suspended in 1 × Annexin V binding buffer and incubated with 1 μL Annexin V-FITC conjugate and 12.5 μL propidium iodide (PI) solution (Santa cruz) for 15 mins. The intensity of the fluorescence was immediately detected using FL1-FITC (Annexin V) and FL2-PI Channels.

### Measurement of ROS production

Huh7 Cells (5 × 10^5^) were seeded in 6-well plates overnight and then treated with MBIC in the absence or presence of N-Acetyl cysteine (NAC) for the indicated times. After treatment, the cells were harvested and stained with 10 μM 2′,7′-Dichlorofluorescin diacetate (DCFH-DA) at 37°C for 30 min in the dark. The fluorescence was measured by flow cytometry (BD, Biosciences, San Jose, CA) using FITC channel.

### Estimation of mitochondrial membrane potential (Δψm)

The change mitochondrial membrane potential was tested using Abcam's TMRE Mitochondrial Membrane Potential Assay Kit (ab113852). The Huh7 cells (7 × 10^3^) were seeded in 96-well plates overnight. Then the cells were incubated with indicated concentrations of MBIC for 12 h. 20 μM FCCP (carbonyl cyaninde 4-(trifluoromethoxy) phenylhydrazone), the positive control, was added to the cell medium 10 mins before staining. 1 μM Tetramethylrhodamine, ethyl ester (TMRE) was added to the cells and incubated for 20 mins 37°C. The TMRE staining was analyzed using a by microplate spectrophotometer (Tecan) at Ex/Em = 549/575 nm.

### Western blotting

Whole-cell extracts were lysed in pre-cold lysis buffer with freshly added protease/phosphatase inhibitors. After 30 mins, the lysates were then spun at 13,300 rpm for 10 minutes and the pellet was discarded. Equal amount of protein were loaded for electrophoresis and then electro-transferred to a nitrocellulose membrane (Biorad, USA), which was blocked with Blocking One (Nacalai Tesque, Inc., Japan), and probed with the primary antibodies of interest overnight at 4°C. The blot was washed, exposed to HRP-conjugated secondary antibodies (Santa Cruz Co, CA) for 1 h, and finally examined by chemiluminescence (ECL, advansta Inc., USA).

### Acute toxicity studies

All animal experiments were performed according to protocols approved by the SingHealth Institutional Animal Use and Care Committee. For the acute toxicity drug study, eight week-old NCr nude female mice (*In vivos*, Singapore) were treated with intraperitoneal injections of 10 mg/kg, 25 mg/kg, or 50 mg/kg of MBIC, and vehicle (0.1% DMSO). The mice were monitored daily for development of any toxic signs such as change in physical appearance, hunched back, increased respiration, arching and rolling, muscle spasm, tremors, cyanosis, stimulation or depression. The body weight changes, food and water intake were monitored daily up to day 8. On day 8, the terminal blood from the mice was collected by cardiac puncture. The liver and kidney functions were studied using the serum. Three main criteria {Alanine transaminase (ALT), Aspartate transaminase (AST)} were examined for liver function, and Blood urea nitrogen (BUN) for kidney function.

### Orthotopic HCC tumor model

For the drug efficacy study, eight week-old NCr nude females were implanted orthotopically with Huh-7-Luc cell-induced tumors. When the bioluminescence signal reached 10^6^, mice were treated with either vehicle (1% DMSO) or 25 mg/kg of MBIC three times per week by intraperitoneal injections for 4 consecutive weeks. The development of the tumor was monitored twice a week by measuring the bioluminescence signals. Mice were euthanized by using CO_2_ inhalation when the humane end-point criteria were met. Primary tumor (liver) and lung tissues were excised, snap-frozen, and stored at –80°C until used for experiments.

### Immunohistochemistry

The control and MBIC-treated mice tissues were fixed with 4% formaldehyde and then dehydrated in graded ethanols and xylene. After embedding with paraffin, the tissue was sliced into 5 μm sections and the slides were baked at 60°C for 2 h. The antigens of the deparaffinised sections were unmasked by boiling in 10 mM sodium citrate buffer (pH 6.0), and microwaved four times for 6 mins each time. After the sections cooled down to room temperature, they were incubated with 3% H_2_O_2_ for 10 mins and blocked by using blocking one (Nacalai Tesque, Inc.) for 1 h. Next, the sections were incubated with the primary antibodies p-JNK (1:50), Ki67 (1:100) and cleaved-caspase3 (1:100) at 4°C overnight. After washing, the slides were incubated with EnVision polymer HRP-Rabbit (DAKO) for 1 h and visualized by using 3, 3′-diamino-benzidine (DAB). Sections were counterstained using Hematoxylin, and cover-slipped after using a mounting medium (Sigma). Images were taken using an Olympus BX51 microscope.

### Statistical analysis

The Student's *t* test or one way ANOVA with Dunnett's Multiple Comparison Test was used for the comparison of measurable variants. All data are presented as means and standard errors of the mean. *p* < 0.05 was considered statistically significant (GraphPad Prism 5.0; Graph Pad Software, CA). The migration and invasion data was quantified by using Image J software.
